# 1-[(Biphenyl-4-yl)(phen­yl)meth­yl]-1*H*-imidazole (bifonazole)

**DOI:** 10.1107/S1600536810037876

**Published:** 2010-09-30

**Authors:** Bernard Van Eerdenbrugh, Phillip E. Fanwick, Lynne S. Taylor

**Affiliations:** a Department of Industrial and Physical Pharmacy, Purdue University, 575 Stadium Mall Drive, West Lafayette, IN 47907, USA; b Laboratory for Pharmacotechnology and Biopharmacy, K.U. Leuven, Gasthuisberg O&N2, Herestraat 49, Box 921, 3000, Leuven, Belgium; c Department of Chemistry, Purdue University, 560 Oval Drive, West Lafayette, IN 47907, USA

## Abstract

In the title compound, C_22_H_18_N_2_, the dihedral angles formed by the imidazole ring with the phenyl ring and the benzene ring of the biphenyl group are 87.02 (5) and 78.20 (4)°, respectively. In the crystal, mol­ecules inter­act through inter­molecular C—H⋯N hydrogen bonds, forming chains parallel to the *b* axis. These chains are further linked into a three-dimensional network by C—H⋯π stacking inter­actions

## Related literature

For a review of the anti­microbial activity of bifonazole and its therapeutic use in superficial mycoses, see: Lackner and Clissold (1989 ▶). 
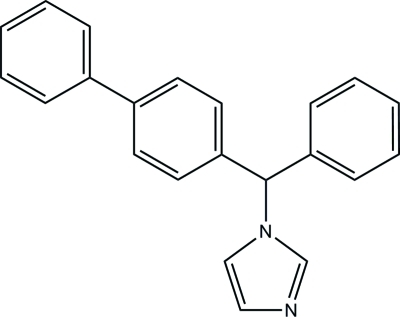


## Experimental

### 

#### Crystal data

C_22_H_18_N_2_
*M* *_r_* = 310.40
Monoclinic, 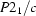

*a* = 7.9737 (7) Å
*b* = 6.2591 (6) Å
*c* = 33.265 (3) Å
β = 93.805 (8)°
*V* = 1656.5 (3) Å^3^
*Z* = 4
Cu *K*α radiation
μ = 0.56 mm^−1^
*T* = 150 K
0.20 × 0.20 × 0.04 mm

#### Data collection

Rigaku RAPID II diffractometer
Absorption correction: multi-scan (*SCALEPACK*; Otwinowski & Minor, 1997 ▶) *T*_min_ = 0.860, *T*_max_ = 0.979
19391 measured reflections
3064 independent reflections
2801 reflections with *I* > 2σ(*I*)
*R*_int_ = 0.030

#### Refinement

*R*[*F* ^2^ > 2σ(*F* ^2^)] = 0.041
*wR*(*F* ^2^) = 0.107
*S* = 1.06
3064 reflections
218 parameters
H-atom parameters constrained
Δρ_max_ = 0.19 e Å^−3^
Δρ_min_ = −0.20 e Å^−3^

### 

Data collection: *CrystalClear* (Rigaku, 2001 ▶); cell refinement: *DENZO*/*SCALEPACK* (Otwinowski & Minor, 1997 ▶); data reduction: *DENZO*/*SCALEPACK*; program(s) used to solve structure: *SIR2004* (Burla *et al.*, 2005 ▶); program(s) used to refine structure: *SHELXL97* (Sheldrick, 2008 ▶); molecular graphics: *ORTEPII* (Johnson, 1976 ▶) and *PLATON* (Spek, 2009 ▶); software used to prepare material for publication: *SHELXL97* and local programs.

## Supplementary Material

Crystal structure: contains datablocks global, I. DOI: 10.1107/S1600536810037876/rz2487sup1.cif

Structure factors: contains datablocks I. DOI: 10.1107/S1600536810037876/rz2487Isup2.hkl

Additional supplementary materials:  crystallographic information; 3D view; checkCIF report

## Figures and Tables

**Table 1:** Hydrogen-bond geometry (Å, °) *Cg*1, *Cg*2 and *Cg*3 are the centroids of the N1/N2/C20–C22, C1–C6 and C14–C19 rings, respectively.

*D*—H⋯*A*	*D*—H	H⋯*A*	*D*⋯*A*	*D*—H⋯*A*
C7—H7⋯N2^i^	1.00	2.45	3.418 (2)	161
C3—H3⋯*Cg*1^ii^	0.95	2.76	3.609 (2)	149
C6—H6⋯*Cg*1^iii^	0.95	2.96	3.900 (3)	171
C18—H18⋯*Cg*2^iv^	0.95	3.01	3.797 (7)	141
C21—H21⋯*Cg*2^v^	0.95	2.76	3.694 (7)	170
C12—H12⋯*Cg*3^vi^	0.95	2.87	3.737 (5)	153

Symmetry codes: (i) 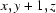; (ii) 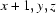; (iii) 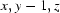; (iv) 
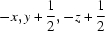
; (v) 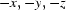; (vi) 
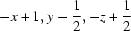
.

**Fig. 1.**
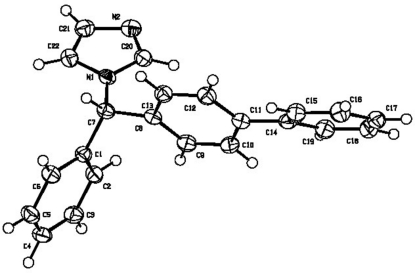
The molecular structure of the title compound, showing 50% probability displacement ellipsoids and the atomic numbering. H atoms are presented as small spheres of arbitrary radius.

## supplementary crystallographic information

### Comment

Bifonazole is a broad-spectrum antifungal agent, mainly used by topical application in the treatment of fungal skin infections, including nail infections (Lackner & Clissold, 1989). In the crystal structure of the racemate, layers of the *R* and *S* enantiomer alternate along the *c* axis. Figure 1 shows the *S* configuration of the chiral center at atom C7. The dihedral angles between the different rings are 26.17 (8)° for the two aromatic rings of the biphenyl group, 101.80 (4)° for the imidazole ring and the benzene ring of the biphenyl group, 62.34 (5)° for the phenyl ring and the benzene ring of the biphenyl group, and 92.98 (5)° for the imidazole ring and the phenyl ring. In the crystal structure, molecules are linked by intermolecular C—H···N hydrogen bonds (Table 1) into chains running parallel to the *b* axis. The chains are further connected by C—H···π stacking interactions to form a three-dimensional network.

### Experimental

A saturated solution of the title compound was prepared by adding an excess of powder to 20 ml of diethyl ether. Subsequent to stirring the suspension overnight, filtration was performed using a 0.2 µm PTFE syringe filter (13 mm, VWR International, LLC, West Chester, PA, USA). The solution was transferred into a 20 ml scintillation vial in 20 ml scintillation vials (Research Products International Corp., Mt. Prospect, IL, USA) and three holes were pierced in the cap of the vial to allow the solvent to slowly evaporate. After one week, all solvent had evaporated and crystals of the title compound were obtained.

### Refinement

H atoms were placed in calculated positions and treated as riding on their parent atoms with C—H = 0.95 Å (aromatic), 1.00 Å (aliphatic) and with *U*_iso_(H) = 1.2 *U*_eq_(C).

### Crystal data

**Table:**

C_22_H_18_N_2_	*F*(000) = 656
*M**_r_* = 310.40	*D*_x_ = 1.245 Mg m^−^^3^
Monoclinic, *P*2_1_/*c*	Cu *K*α radiation, λ = 1.54184 Å
Hall symbol: -P 2ybc	Cell parameters from 3380 reflections
*a* = 7.9737 (7) Å	θ = 2–71°
*b* = 6.2591 (6) Å	µ = 0.56 mm^−^^1^
*c* = 33.265 (3) Å	*T* = 150 K
β = 93.805 (8)°	Plate, colourless
*V* = 1656.5 (3) Å^3^	0.20 × 0.20 × 0.04 mm
*Z* = 4	

### Data collection

**Table:**

Rigaku Rapid II diffractometer	2801 reflections with *I* > 2σ(*I*)
confocal optics	*R*_int_ = 0.030
ω scans	θ_max_ = 71.8°, θ_min_ = 2.7°
Absorption correction: multi-scan (*SCALEPACK*; Otwinowski & Minor, 1997)	*h* = 0→9
*T*_min_ = 0.860, *T*_max_ = 0.979	*k* = 0→7
19391 measured reflections	*l* = −40→40
3064 independent reflections	

### Refinement

**Table:**

Refinement on *F*^2^	H-atom parameters constrained
Least-squares matrix: full	*w* = 1/[σ^2^(*F*_o_^2^) + (0.0501*P*)^2^ + 0.7321*P*] where *P* = (*F*_o_^2^ + 2*F*_c_^2^)/3
*R*[*F*^2^ > 2σ(*F*^2^)] = 0.041	(Δ/σ)_max_ < 0.001
*wR*(*F*^2^) = 0.107	Δρ_max_ = 0.19 e Å^−^^3^
*S* = 1.06	Δρ_min_ = −0.20 e Å^−^^3^
3064 reflections	Extinction correction: *SHELXL97* (Sheldrick, 2008)
218 parameters	Extinction coefficient: 0.20E-02
0 restraints	

### Special details

**Table:**

Geometry. All e.s.d.'s (except the e.s.d. in the dihedral angle between two l.s. planes) are estimated using the full covariance matrix. The cell e.s.d.'s are taken into account individually in the estimation of e.s.d.'s in distances, angles and torsion angles; correlations between e.s.d.'s in cell parameters are only used when they are defined by crystal symmetry. An approximate (isotropic) treatment of cell e.s.d.'s is used for estimating e.s.d.'s involving l.s. planes.
Refinement. Outlier data were removed using a local program based on the method of Prince and Nicholson.Refinement on *F*^2^ for ALL reflections except for 0 with very negative *F*^2^ or flagged by the user for potential systematic errors. Weighted *R*-factors *wR* and all goodnesses of fit *S* are based on *F*^2^, conventional *R*-factors *R* are based on *F*, with *F* set to zero for negative *F*^2^. The observed criterion of *F*^2^ > σ(*F*^2^) is used only for calculating R_factor_obs *etc*. and is not relevant to the choice of reflections for refinement. *R*-factors based on *F*^2^ are statistically about twice as large as those based on *F*, and *R*- factors based on ALL data will be even larger.

### Fractional atomic coordinates and isotropic or equivalent isotropic displacement parameters (Å^2^)

**Table:**

	*x*	*y*	*z*	*U*_iso_*/*U*_eq_	
N1	0.15014 (12)	0.10641 (19)	0.07667 (3)	0.0251 (3)	
N2	0.26374 (14)	−0.2162 (2)	0.07143 (3)	0.0316 (3)	
C1	−0.10284 (15)	0.3342 (2)	0.07936 (4)	0.0247 (3)	
C2	−0.21816 (16)	0.1678 (2)	0.08086 (4)	0.0277 (3)	
C3	−0.38706 (16)	0.2022 (2)	0.06900 (4)	0.0313 (3)	
C4	−0.44051 (16)	0.4013 (3)	0.05515 (4)	0.0322 (3)	
C5	−0.32601 (17)	0.5661 (2)	0.05271 (4)	0.0324 (3)	
C6	−0.15719 (16)	0.5331 (2)	0.06485 (4)	0.0291 (3)	
C7	0.08225 (15)	0.3066 (2)	0.09321 (4)	0.0247 (3)	
C8	0.12033 (15)	0.3155 (2)	0.13881 (4)	0.0245 (3)	
C9	0.02747 (16)	0.2044 (2)	0.16633 (4)	0.0288 (3)	
C10	0.07544 (16)	0.2074 (2)	0.20723 (4)	0.0290 (3)	
C11	0.21664 (15)	0.3221 (2)	0.22243 (4)	0.0262 (3)	
C12	0.30588 (16)	0.4380 (2)	0.19481 (4)	0.0288 (3)	
C13	0.25869 (16)	0.4334 (2)	0.15381 (4)	0.0276 (3)	
C14	0.27240 (15)	0.3177 (2)	0.26618 (4)	0.0266 (3)	
C15	0.36722 (17)	0.4845 (2)	0.28421 (4)	0.0318 (3)	
C16	0.42239 (18)	0.4754 (3)	0.32482 (4)	0.0350 (3)	
C17	0.38402 (17)	0.3018 (2)	0.34818 (4)	0.0319 (3)	
C18	0.28887 (17)	0.1358 (3)	0.33100 (4)	0.0342 (3)	
C19	0.23430 (17)	0.1436 (2)	0.29038 (4)	0.0324 (3)	
C20	0.21208 (16)	−0.0692 (2)	0.09636 (4)	0.0283 (3)	
C21	0.23282 (16)	−0.1299 (2)	0.03361 (4)	0.0316 (3)	
C22	0.16304 (17)	0.0681 (2)	0.03627 (4)	0.0314 (3)	
H2	−0.1817	0.0306	0.0900	0.033*	
H3	−0.4657	0.0888	0.0704	0.038*	
H4	−0.5558	0.4246	0.0473	0.039*	
H5	−0.3624	0.7018	0.0428	0.039*	
H6	−0.0789	0.6467	0.0632	0.035*	
H7	0.1445	0.4277	0.0814	0.030*	
H9	−0.0694	0.1261	0.1569	0.035*	
H10	0.0110	0.1299	0.2253	0.035*	
H12	0.4002	0.5210	0.2042	0.035*	
H13	0.3221	0.5123	0.1357	0.033*	
H15	0.3942	0.6052	0.2686	0.038*	
H16	0.4870	0.5896	0.3366	0.042*	
H17	0.4225	0.2962	0.3758	0.038*	
H18	0.2609	0.0167	0.3469	0.041*	
H19	0.1699	0.0286	0.2789	0.039*	
H20	0.2177	−0.0849	0.1248	0.034*	
H21	0.2567	−0.1985	0.0092	0.038*	
H22	0.1300	0.1609	0.0146	0.038*	

### Atomic displacement parameters (Å^2^)

**Table:**

	*U*^11^	*U*^22^	*U*^33^	*U*^12^	*U*^13^	*U*^23^
N1	0.0191 (5)	0.0280 (6)	0.0278 (5)	0.0017 (4)	−0.0006 (4)	0.0008 (4)
N2	0.0282 (6)	0.0301 (7)	0.0369 (6)	0.0021 (5)	0.0042 (5)	−0.0010 (5)
C1	0.0227 (6)	0.0281 (8)	0.0231 (6)	0.0019 (5)	0.0004 (5)	−0.0019 (5)
C2	0.0263 (6)	0.0274 (8)	0.0293 (6)	0.0016 (5)	−0.0002 (5)	0.0023 (5)
C3	0.0239 (6)	0.0358 (9)	0.0342 (7)	−0.0037 (6)	0.0024 (5)	0.0001 (6)
C4	0.0217 (6)	0.0417 (9)	0.0331 (7)	0.0063 (6)	0.0002 (5)	−0.0025 (6)
C5	0.0309 (7)	0.0316 (9)	0.0341 (7)	0.0090 (6)	−0.0016 (5)	0.0006 (6)
C6	0.0277 (7)	0.0270 (8)	0.0324 (7)	0.0007 (5)	0.0003 (5)	−0.0004 (6)
C7	0.0213 (6)	0.0220 (7)	0.0307 (7)	−0.0002 (5)	0.0012 (5)	0.0004 (5)
C8	0.0213 (6)	0.0229 (7)	0.0293 (6)	0.0039 (5)	0.0006 (5)	−0.0006 (5)
C9	0.0213 (6)	0.0305 (8)	0.0343 (7)	−0.0044 (5)	−0.0003 (5)	−0.0015 (6)
C10	0.0233 (6)	0.0326 (8)	0.0314 (7)	−0.0033 (5)	0.0039 (5)	0.0017 (6)
C11	0.0218 (6)	0.0268 (8)	0.0301 (7)	0.0022 (5)	0.0010 (5)	−0.0013 (5)
C12	0.0239 (6)	0.0290 (8)	0.0330 (7)	−0.0040 (5)	−0.0015 (5)	−0.0006 (5)
C13	0.0241 (6)	0.0269 (8)	0.0318 (7)	−0.0017 (5)	0.0019 (5)	0.0028 (5)
C14	0.0197 (6)	0.0308 (8)	0.0296 (7)	0.0012 (5)	0.0026 (5)	−0.0009 (5)
C15	0.0299 (7)	0.0335 (8)	0.0319 (7)	−0.0032 (6)	0.0011 (5)	0.0007 (6)
C16	0.0327 (7)	0.0381 (9)	0.0337 (7)	−0.0041 (6)	−0.0016 (6)	−0.0051 (6)
C17	0.0270 (6)	0.0408 (9)	0.0276 (7)	0.0029 (6)	−0.0001 (5)	−0.0020 (6)
C18	0.0305 (7)	0.0398 (9)	0.0325 (7)	−0.0019 (6)	0.0041 (5)	0.0055 (6)
C19	0.0281 (7)	0.0360 (9)	0.0328 (7)	−0.0052 (6)	0.0003 (5)	−0.0006 (6)
C20	0.0255 (6)	0.0288 (8)	0.0306 (7)	0.0018 (5)	0.0007 (5)	0.0020 (5)
C21	0.0281 (7)	0.0359 (9)	0.0308 (7)	0.0006 (6)	0.0031 (5)	−0.0045 (6)
C22	0.0301 (7)	0.0370 (9)	0.0267 (6)	0.0041 (6)	−0.0003 (5)	0.0005 (6)

### Geometric parameters (Å, °)

**Table:**

N1—C20	1.3560 (17)	C10—C11	1.4011 (18)
N1—C22	1.3758 (17)	C10—H10	0.9500
N1—C7	1.4854 (17)	C11—C12	1.4012 (19)
N2—C20	1.3226 (18)	C11—C14	1.4935 (18)
N2—C21	1.3765 (18)	C12—C13	1.3907 (18)
C1—C2	1.3921 (19)	C12—H12	0.9500
C1—C6	1.3940 (19)	C13—H13	0.9500
C1—C7	1.5263 (16)	C14—C19	1.400 (2)
C2—C3	1.3947 (18)	C14—C15	1.4005 (19)
C2—H2	0.9500	C15—C16	1.3939 (19)
C3—C4	1.386 (2)	C15—H15	0.9500
C3—H3	0.9500	C16—C17	1.381 (2)
C4—C5	1.384 (2)	C16—H16	0.9500
C4—H4	0.9500	C17—C18	1.387 (2)
C5—C6	1.3949 (19)	C17—H17	0.9500
C5—H5	0.9500	C18—C19	1.3928 (19)
C6—H6	0.9500	C18—H18	0.9500
C7—C8	1.5280 (17)	C19—H19	0.9500
C7—H7	1.0000	C20—H20	0.9500
C8—C13	1.3921 (18)	C21—C22	1.364 (2)
C8—C9	1.4000 (19)	C21—H21	0.9500
C9—C10	1.3887 (18)	C22—H22	0.9500
C9—H9	0.9500		
			
C20—N1—C22	106.39 (11)	C10—C11—C12	117.35 (12)
C20—N1—C7	129.43 (11)	C10—C11—C14	121.48 (12)
C22—N1—C7	124.18 (11)	C12—C11—C14	121.16 (12)
C20—N2—C21	104.82 (12)	C13—C12—C11	121.09 (12)
C2—C1—C6	119.30 (11)	C13—C12—H12	119.50
C2—C1—C7	122.18 (12)	C11—C12—H12	119.50
C6—C1—C7	118.52 (12)	C12—C13—C8	121.25 (12)
C1—C2—C3	120.16 (13)	C12—C13—H13	119.40
C1—C2—H2	119.90	C8—C13—H13	119.40
C3—C2—H2	119.90	C19—C14—C15	117.75 (12)
C4—C3—C2	120.16 (13)	C19—C14—C11	120.86 (12)
C4—C3—H3	119.90	C15—C14—C11	121.38 (12)
C2—C3—H3	119.90	C16—C15—C14	120.74 (14)
C5—C4—C3	120.03 (12)	C16—C15—H15	119.60
C5—C4—H4	120.00	C14—C15—H15	119.60
C3—C4—H4	120.00	C17—C16—C15	120.66 (14)
C4—C5—C6	120.02 (13)	C17—C16—H16	119.70
C4—C5—H5	120.00	C15—C16—H16	119.70
C6—C5—H5	120.00	C16—C17—C18	119.52 (13)
C1—C6—C5	120.30 (13)	C16—C17—H17	120.20
C1—C6—H6	119.80	C18—C17—H17	120.20
C5—C6—H6	119.80	C17—C18—C19	120.02 (14)
N1—C7—C1	110.62 (10)	C17—C18—H18	120.00
N1—C7—C8	110.18 (10)	C19—C18—H18	120.00
C1—C7—C8	114.84 (10)	C18—C19—C14	121.31 (13)
N1—C7—H7	106.90	C18—C19—H19	119.30
C1—C7—H7	106.90	C14—C19—H19	119.30
C8—C7—H7	106.90	N2—C20—N1	112.32 (12)
C13—C8—C9	118.03 (12)	N2—C20—H20	123.80
C13—C8—C7	118.39 (11)	N1—C20—H20	123.80
C9—C8—C7	123.53 (11)	C22—C21—N2	110.27 (12)
C10—C9—C8	120.71 (12)	C22—C21—H21	124.90
C10—C9—H9	119.60	N2—C21—H21	124.90
C8—C9—H9	119.60	C21—C22—N1	106.21 (12)
C9—C10—C11	121.53 (12)	C21—C22—H22	126.90
C9—C10—H10	119.20	N1—C22—H22	126.90
C11—C10—H10	119.20		
			
C6—C1—C2—C3	−1.77 (19)	C10—C11—C12—C13	−1.9 (2)
C7—C1—C2—C3	178.09 (12)	C14—C11—C12—C13	176.77 (12)
C1—C2—C3—C4	0.9 (2)	C11—C12—C13—C8	0.6 (2)
C2—C3—C4—C5	0.6 (2)	C9—C8—C13—C12	1.3 (2)
C3—C4—C5—C6	−1.1 (2)	C7—C8—C13—C12	−176.27 (12)
C2—C1—C6—C5	1.24 (19)	C10—C11—C14—C19	25.82 (19)
C7—C1—C6—C5	−178.63 (12)	C12—C11—C14—C19	−152.84 (13)
C4—C5—C6—C1	0.2 (2)	C10—C11—C14—C15	−155.53 (13)
C20—N1—C7—C1	−116.94 (13)	C12—C11—C14—C15	25.82 (19)
C22—N1—C7—C1	63.98 (15)	C19—C14—C15—C16	0.5 (2)
C20—N1—C7—C8	11.12 (17)	C11—C14—C15—C16	−178.19 (12)
C22—N1—C7—C8	−167.95 (11)	C14—C15—C16—C17	−0.3 (2)
C2—C1—C7—N1	46.28 (16)	C15—C16—C17—C18	−0.3 (2)
C6—C1—C7—N1	−133.85 (12)	C16—C17—C18—C19	0.7 (2)
C2—C1—C7—C8	−79.20 (15)	C17—C18—C19—C14	−0.5 (2)
C6—C1—C7—C8	100.67 (14)	C15—C14—C19—C18	−0.1 (2)
N1—C7—C8—C13	98.12 (14)	C11—C14—C19—C18	178.57 (12)
C1—C7—C8—C13	−136.18 (12)	C21—N2—C20—N1	−0.11 (15)
N1—C7—C8—C9	−79.26 (15)	C22—N1—C20—N2	0.13 (15)
C1—C7—C8—C9	46.44 (18)	C7—N1—C20—N2	−179.07 (11)
C13—C8—C9—C10	−1.8 (2)	C20—N2—C21—C22	0.04 (15)
C7—C8—C9—C10	175.57 (12)	N2—C21—C22—N1	0.03 (15)
C8—C9—C10—C11	0.5 (2)	C20—N1—C22—C21	−0.09 (14)
C9—C10—C11—C12	1.4 (2)	C7—N1—C22—C21	179.16 (11)
C9—C10—C11—C14	−177.33 (12)		

### Hydrogen-bond geometry (Å, °)

**Table:**

*Cg*1, *Cg*2 and *Cg*3 are the centroids of the N1/N2/C20–C22, C1–C6 and C14–C19 rings, respectively.

**Table:**

*D*—H···*A*	*D*—H	H···*A*	*D*···*A*	*D*—H···*A*
C7—H7···N2^i^	1.00	2.45	3.418 (2)	161
C3—H3···Cg1^ii^	0.95	2.76	3.609 (2)	149
C6—H6···Cg1^iii^	0.95	2.96	3.900 (3)	171
C18—H18···Cg2^iv^	0.95	3.01	3.797 (7)	141
C21—H21···Cg2^v^	0.95	2.76	3.694 (7)	170
C12—H12···Cg3^vi^	0.95	2.87	3.737 (5)	153

Symmetry codes: (i) *x*, *y*+1, *z*; (ii) *x*+1, *y*, *z*; (iii) *x*, *y*−1, *z*; (iv) −*x*, *y*+1/2, −*z*+1/2; (v) −*x*, −*y*, −*z*; (vi) −*x*+1, *y*−1/2, −*z*+1/2.
